# LeafScope: A Portable High-Resolution Multispectral Imager for In Vivo Imaging Soybean Leaf

**DOI:** 10.3390/s20082194

**Published:** 2020-04-13

**Authors:** Liangju Wang, Yunhong Duan, Libo Zhang, Jialei Wang, Yikai Li, Jian Jin

**Affiliations:** 1 Department of Agricultural and Biological Engineering, Purdue University, 225 S. University St., West Lafayette, IN 47907, USA; wang3335@purdue.edu (L.W.); duan70@purdue.edu (Y.D.); zhan2693@purdue.edu (L.Z.); wang2700@purdue.edu (J.W.); 2 School of Industrial Engineering, Purdue University, 315 Grant St., West Lafayette, IN 47907, USA; li2039@purdue.edu

**Keywords:** plant phenotyping, handheld sensor, soybean leaf, multispectral imaging, high-resolution, leaf morphological features, leaf venation

## Abstract

Portable devices for measuring plant physiological features with their isolated measuring chamber are playing an increasingly important role in plant phenotyping. However, currently available commercial devices of this type, such as soil plant analysis development (SPAD) meter and spectrometer, are dot meters that only measure a small region of the leaf, which does not perfectly represent the highly varied leaf surface. This study developed a portable and high-resolution multispectral imager (named LeafScope) to in-vivo image a whole leaf of dicotyledon plants while blocking the ambient light. The hardware system is comprised of a monochrome camera, an imaging chamber, a lightbox with different bands of light-emitting diodes (LEDs) array, and a microcontroller. During measuring, the device presses the leaf to lay it flat in the imaging chamber and acquires multiple images while alternating the LED bands within seconds in a certain order. The results of an experiment with soybean plants clearly showed the effect of nitrogen and water treatments as well as the genotype differences by the color and morphological features from image processing. We conclude that the low cost and easy to use LeafScope can provide promising imaging quality for dicotyledon plants, so it has great potential to be used in plant phenotyping.

## 1. Introduction

Leaf is a crucial organ in plant energy capture and carbon conversion [1,2]. It has been reported that the color, morphology, and venation pattern of plant leaf contain useful information to improve phenotyping quality. Leaf color, especially the color distribution, is used to detect nutrient deficiencies and diseases of plants [3,4]. For instance, RGB images of soybean leaves are used to predict stress status and detect soybean aphids [5,6,7]. Leaf morphological features, such as area, perimeter, and roundness, are also studied for plant physiological phenotyping. For instance, leaf morphology is used to evaluate plant drought resistance in arid ecosystems [8]. Leaf venation is reported to relate to leaf mechanical stability and the transport of water and carbohydrates [9]. Moreover, the venation becomes increasingly important in plant phenotyping while more and more venation extraction algorithms appear, such as LEAF GUI, PhenoVein, and NET [10,11,12,13,14,15].

The traditional way of measuring the leaf’s physiological features is to harvest the leaf from the plant and then send it for lab analysis [16,17,18]. However, this method is destructive to the plant and not efficient. In recent years, many non-destructive plant phenotyping methods are developed based on imaging technology to monitor plant conditions, such as remote imaging with a UAV, proximal imaging with the mobile platform in the field, or phenotyping facility in a greenhouse [19,20,21,22,23]. However, these imaging systems monitor the plant conditions in plot level or whole canopy level, while the detailed information of an individual leaf, including color distribution and leaf venation, cannot be obtained. Currently, the reported portable devices to measure leaf in vivo can be categorized into two kinds, dot meters and whole leaf imagers. Dot meters measure one small region on a leaf per capture while whole leaf imagers capture the whole leaf image. The dot meter has been commercialized and used widely in plant phenotyping. Two of the most popular devices are the Minolta SPAD® meter (Minolta Co., Ltd., Osaka, Honshu, Japan) and spectrometer, such as ASD FieldSpec 4 (Malvern Panalytical Ltd., Malvern, UK). Both of them measure the color or spectrum of a small region on the leaf. Still, the information of color distribution on the leaf surface, morphology, and venation pattern is undetectable with a dot meter [24,25,26]. The whole leaf imager is more informative than the dot meter. Currently, there are few professional leaf imagers reported. The most common way to image a whole leaf is to use a smartphone or RGB camera to capture the leaf image directly [27]. However, this method cannot block the ambient light and suffers from the changing leaf slopes, which impacts the imaging results [28]. Moreover, two whole leaf portable imagers with an imaging chamber were reported for monocotyledon plants whose leaves are long and narrow. Wang et al. developed a whole leaf hyperspectral imaging device, called LeafSpec, for corn plants to measure the nitrogen content and relative water content [29]. Zhang et al. reported a portable and low-cost multispectral corn leaf scanner to measure nitrogen contents and leaf area [30]. However, these existing whole leaf imaging devices are not suitable for dicotyledon plants whose leaves are short and broad.

To obtain high-quality and comprehensive imaging signals of dicotyledon plant leaves in vivo, we developed a new portable device, named LeafScope. The device captured the multispectral image of a whole leaf with high-resolution nondestructively. Its ability to measure the leaf color, morphology, and venation pattern was evaluated in greenhouse assays with soybean plants, a representative of dicotyledon plants. Furthermore, the device was compared against SPAD meter in its ability to measure the nitrogen contents of plants of different genotypes under different treatments.

## 2. Development of LeafScope

The LeafScope captures a high-resolution multispectral image of the whole leaf by gently grasping the living leaf inside the device’s imaging chamber. The image is stored and processed in the on-board microcontroller in real-time. The processed result is stored in the SD card mounted on the device for downloading and future analysis. A part of the processed data can also be transferred through Bluetooth to a smartphone application (App) “LeafSpec” for real-time interaction and view. If needed, the data can also be uploaded to the Purdue geographic information system (GIS) server with global positioning system (GPS) information by the LeafSpec App [29]. The entire procedure of taking one multispectral image of a leaf takes around 15 s. The hardware, workflow, data processing, and LeafSpec App are explained in detail below.

### 2.1. Hardware Configuration

**Figure 1:**
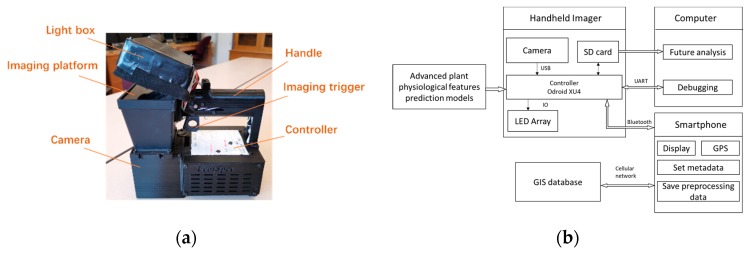
Handheld multispectral imager LeafScope. (**a**) Photograph of LeafScope. (**b**) The data flow and communication in LeafScope system.

LeafScope (Figure 1a) images the leaf to obtain the transmittance multispectral image of four bands, 405, 560, 660, and 880 nm, from blue to near-infrared (NIR). The device’s total weight is about 0.6 kg with length, width, and height of 225, 140, and 230 mm, respectively. The dimension of the leaf it can image is up to 124 × 80 mm, which is almost the dimension of the imaging chamber of the device. The hardware consists of four main components: a monochromatic camera (BFLY-U3-23S6M-C, FLIR Integrated Imaging Solutions Inc., Richmond, BC, Canada) with a C-mount lens (V0814-MP, Computar®, Tokyo, Japan), an imaging chamber, a lightbox, and an ARM® based microcontroller (ODROID XU4, Hardkernel Co., Ltd, Gyeonggi, South Korea). The device is powered by a portable power bank (FBA_YB1206000, TalentCell, Guangdong, China).

To capture a high-resolution multispectral image of the leaf, a camera with high resolution and broad dynamic range was selected. The parameters of the camera are shown in Table 1. The lens with an 8 mm focal length was selected to decrease the distance between the camera and the leaf and achieve a small volume device. ODROID XU4 is selected as the microcontroller of LeafScope due to the low cost and higher image processing performance compared with Raspberry Pi. The microcontroller acquires images from the camera, processes the data, and communicates with the “LeafSpec” APP. The selection of the bands of LEDs was based on the application of LeafScope and its designed scale and structure. As the visible and NIR reflectance contained tremendous information on plant health, especially the chlorophyll and nitrogen status, the sensitive wavelength range of LeafScope was selected from visible to NIR. Additionally, to simplify the structure of the LED board and reduce the cost, we installed four types of LED, representing blue, green, red, and near-infrared bands, respectively, inside the lightbox. The selected bands of LEDs could be changed by redesigning the LED PCB board based on the application of LeafScope, e.g., red and NIR LEDs could be selected if only the normalized difference vegetation index (NDVI) was required. In this study, four types of LEDs are selected as light sources, including 405 nm blue LEDs (SM0603UV-400, Bivar Inc., Irvine, CA, US), 560 nm green LEDs (SML-H12P8TT86, ROHM Semiconductor, Kyoto, Japan), 660 nm red LEDs (SML-LXF0805SRD-TR, Lumex Inc., Carol Stream, IL, US), and 880 nm NIR LEDs (APT2012SF4C-PRV, Kingbright, Taipei, Taiwan, China). The LEDs are distributed on the LED board in groups. Each group includes all four types of LEDs so to mix the different color bands well and provide a uniform lighting distribution. Consequently, there are 48 groups and 192 LEDs in total distributed on the LED board with a dimension of 140 mm × 90 mm. A white Teflon plate with a thickness of 2.54 mm is mounted below the LED board to diffuse the light.

In the device, all LEDs were approximately $50, the camera and its lens were close to $900, and the microcontroller was around $50. Including these critical components, the gross material cost of LeafScope was within $1000. The cost was much lower than Minolta SPAD meter (approximately $2500) and ASD FieldSpec 4 (roughly $5000). The total cost can be lower if we optimize the configuration of the device in the future. For instance, the camera PHX064S-MC (LUCID Vision Labs, Inc., Richmond, Canada) with a lower price (approximately $325) also meets our needs.

### 2.2. Workflow of LeafScope

The data flow and communication in the LeafScope system is illustrated in Figure 1b. The camera collects four monochrome images of a leaf in the imaging chamber while turning on each band of LEDs in order of NIR, red, green, and blue band. Then, the images are processed by the microcontroller. Firstly, the four monochrome images are combined to form a multispectral image. Secondly, the images are calibrated and segmented. Lastly, the leaf’s color-based and morphological features are calculated. Users can input various metadata for each image, such as project name and plant genotype, into the smartphone App. The metadata will also be transmitted through Bluetooth to the device’s microcontroller. The processed data and metadata are saved in the device’s storage and can be downloaded for further analysis. A part of processed data is also sent and showed on the LeafSpec App and could be uploaded to the Purdue GIS database to be viewed as geo-referenced measurements on the map. All of these procedures are conducted automatically in real-time. The controlling programs in the device were developed in Python language.

### 2.3. Image Processing

**Figure 2:**
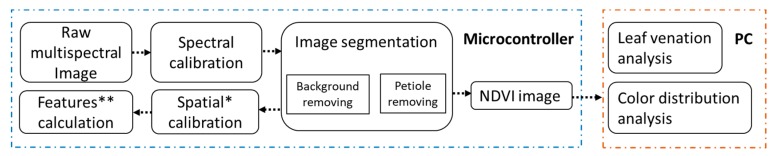
Image processing on microcontroller and computer (PC). Leaf venation analysis and color distribution analysis on PC are elaborated in Section 3. * The image is calibrated with the calibration parameters hardcoded in the microcontroller. ** Features include color features and morphological features.

The output of the LeafScope contains multispectral images, color-based features, morphological features, and metadata input from the App. The former three types of data are automatically processed and generated with the algorithms embedded in the microcontroller. All image processing algorithms are programmed based on OpenCV in Python language, and the procedure is shown in Figure 2. Leaf venation and color distribution analyses were conducted on a personal computer (PC).

#### 2.3.1. Spectral Calibration

To remove the effect of nonuniform light and dark noises of the camera, the multispectral image of the leaf is calibrated by the white and dark reference image with Equation (1),

(1) $$I_{cal}=\frac{I_{raw}-I_{dark}}{I_{white}-I_{dark}}$$

where *I_raw_* is the raw multispectral image of the leaf while turning the specific band of LEDs on, *I_dark_* is the multispectral image without leaf while all LEDs are off, and *I_white_* is the multispectral image without leaf while turning the specific band of LEDs on.

#### 2.3.2. Image Segmentation

As the characteristic of the plants that highly absorb red light and highly reflect NIR light distinguished from that of the background, NDVI is used to segment the multispectral image and calculated by the image of red and NIR band (Equation (2)),

(2) $$NDVI_{m}=\frac{I_{i}-I_{r}}{I_{i}+I_{r}}$$

where *I_i_* and *I_r_* are the calibrated intensity values of NIR and red band image, respectively. The segmented multispectral image is obtained with Equation (3),

(3) $$i_{x,y\;}=\{\begin{array}{c} p_{x,y},n_{x,y}\geq t\\ 0,\;\;n_{x,y}<t \end{array}$$

where *i_x,y_* is the segmented pixel value of the multispectral image, *p_x,y_* is the pixel value of the multispectral image after calibrated, *n_x,y_* is the pixel value of the NDVI image, *(x,y)* is the index of the pixel in the image, and *t* is the threshold value. In the imaging chamber, the background of the image is a white Teflon plate, whose NDVI value is less than 0.2. Thus, 0.2 is selected as the threshold value *t* in this study. However, the threshold value is changeable if necessary; for example, the threshold value could be increased if the yellow disease spots on the leaf are required to be removed.

**Figure 3:**
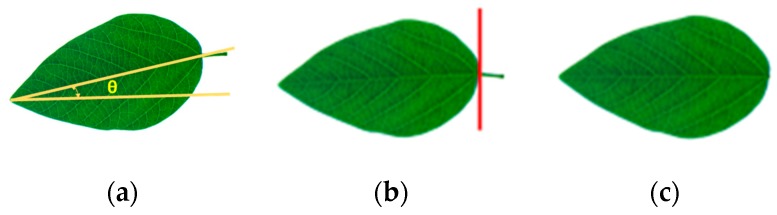
The procedure to remove the petiole in the image. (**a**) Raw image, θ is the angle between the major leaf axis and the image’s *x*-axis. (**b**) Rotated image with the cutting line. (**c**) Result image.

As the leaf is on the plant while imaged by LeafScope, the petiole is also imaged. The procedure to remove the petiole is shown in Figure 3. In the image, the petiole is almost along the major leaf axis due to the placing direction during measurement (Figure 3a). After the major axis is detected by fitting an ellipse on the leaf contour, the angle θ between the major leaf axis and the image’s *x*-axis is calculated. The image is rotated with θ until the major leaf axis is horizontal (Figure 3b). Leaf’s width distribution along the major leaf axis is obtained by counting the leaf’s pixels of each column. The first column on the right side of the leaf is selected as the cutting line, where the leaf width is less than a threshold value *t_petiole_* (Figure 3b). The threshold value *t_petiole_* is 50 in this study, but adjustable when the device is used for other species, for example, apple leaf. All pixels on the right of the cutting line are finally set to zero (Figure 3c).

#### 2.3.3. Color-based and Morphological Features Extraction

The multispectral image can be processed in many different ways depending on the specific phenotyping application. However, LeafScope provides pre-installed onboard imaging processing software to provide some common color-based features and morphological features. The color-based features of a leaf include averaged intensity of each band, averaged NDVI value, and NDVI image. The averaged intensity of each band is calculated by averaging all leaf’s pixels intensity of each band image. An NDVI image is obtained by calculating the NDVI of each pixel on the leaf with Equation (2). Then, by averaging all pixels of the NDVI image, the averaged NDVI value is obtained.

To measure leaf morphological features with high accuracy, the camera is required to be calibrated in the spatial dimension. A checkboard is imaged 12 times from different angles by the camera. With these images, the camera is calibrated with the Matlab Camera Calibration Toolbox (Matlab 2019a), and the calibration parameters, including camera intrinsic matrix and distortion coefficients, are obtained. Besides, the distance between the imaging platform and optical center is also measured. The calibration parameters and distance are hardcoded inside LeafScope for calibrating each leaf image. During imaging, LeafsScope captures a raw image of the leaf, then applies spectral calibration, segmentation, spatial calibration on it (Figure 2). Then, the leaf’s contour is obtained in the unit of mm based on the segmented image. Leaf’s area, perimeter, major axis length, and minor axis length are calculated based on the contour. The major and minor axes length is obtained from the ellipse fitted on the contour. The roundness is calculated with Equation (4),

(4) $$R=\frac{4\pi A}{P^{2}}$$

where *A* is the area of the contour, and *P* is the perimeter of the contour.

### 2.4. LeafSpec App

**Figure 4:**
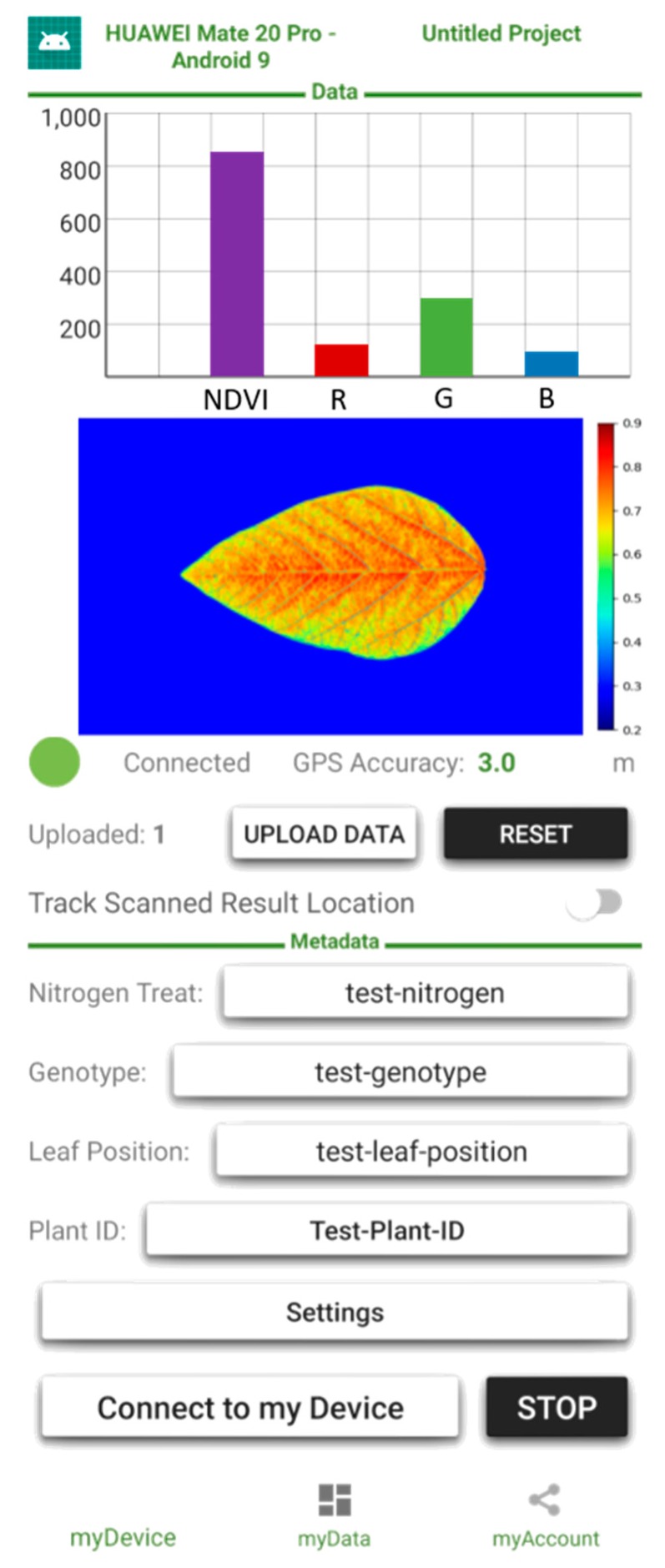
The LeafSpec App on Android.

LeafSpec App was developed for interaction with a series of portable leaf imaging devices developed by Purdue sensor engineers. The App can be used for examining the measurements in real-time, when a smartphone with the App is connected with the device through Bluetooth. With the current LeafScope version, the color-based features are sent to LeafSpec App. As illustrated in Figure 4, the averaged intensity of each band, the averaged NDVI value, and the NDVI image are shown as a bar chart and heatmap on the App interface, respectively. Metadata, including project name, plant ID, leaf location on the plant, genotype, and treatment, is input during the measurement. The resolution of the NDVI image is resized to 192 × 120 in the LeafScope device to decrease the transfer time to the smartphone. The App is for Android smartphones and developed in Android Studio in Java language.

## 3. Test of LeafScope

Soybean plants with different treatments were planted in the greenhouse and measured with the LeafScope. The color-based, morphological, and venation features of leaves were analyzed. The device was also compared with the SPAD meter to test its ability of detecting plant nitrogen contents. The test materials and methods, including plant samples, data acquisition, and statistical analysis, are explained in detail below.

### 3.1. Plant Samples

Two soybean genotypes, NAM_IA and Harosoy, were grown in the Purdue Horticulture Plant Growth Facility (40°25’15.4"N, 86°54’50.4"W). The average temperature in the greenhouse was 25 C°, and the photoperiod provided by the complementary lights was 14 h from 8 am to 10 pm. The lighting intensity was 100 micromol/m2/second PAR in the growing area. The soybean seeds were sowed into cell flats filled with the Berger BM2 germination mix. The seedlings were transplanted into the pots (C300S, Nursery Supplies Inc., Chambersburg, PA, US) filled with BM8 water-saving mix ten days later. The top diameter and height of the pot were about 165 mm and 165 mm, respectively. Each pot contained one seedling. Two different water regimes (well-watered and drought-stressed) and two nitrogen levels (low and high) were applied to the plants. The high nitrogen (HN) and low nitrogen (LN) treatments were achieved with 300 ppm fertilizers and without fertilizers, respectively. When irrigating, each pot for well-watered (WW) plant was watered to excess and allowed to drain, whereas the drought-stressed (DS) plants were watered 500 mL. All plants were irrigated once every two days, and the DS plants were stopped irrigating two days before imaging. To minimize the effects of the microenvironment in the greenhouse, the soybean plants were distributed randomly. The experiment, consisting of eight groups (two genotypes × two water regimes × two N levels), was replicated five times in a full factorial design, thereby giving a total of 40 plants. 

### 3.2. Data Acquisition

**Figure 5:**
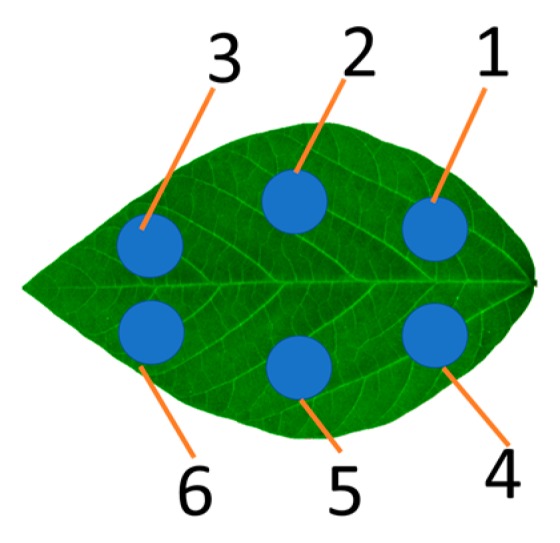
The six dots measured by SPAD meter.

The experiment was carried out in the greenhouse on March 25, 2019, from 3:30 PM to 5:30 PM. The plants were at the R1 stage (Beginning bloom). The middle leaves at the third expanded node from the top were imaged in vivo by LeafScope. The leaf was put into the imaging chamber to block the ambient light while imaging. The smartphone, Huawei® Mate 20 (Huawei Technologies Co., Ltd., Shenzhen, Guangdong China) with the Android 9.0 operating system, was connected with LeafScope for data visualization and metadata input. Also, six dots on the leaf were measured with SPAD-502Plus meter (Konica Minolta Sensing Americas, Inc., Ramsey, NJ, USA). These measured dots’ positions are shown in Figure 5. The captured multispectral images, color-based features, and morphological features were finally downloaded from the device to the computer for statistical analysis. The recorded SPAD values were written into a table file.

### 3.3. Color Distribution across the Leaf

**Figure 6:**
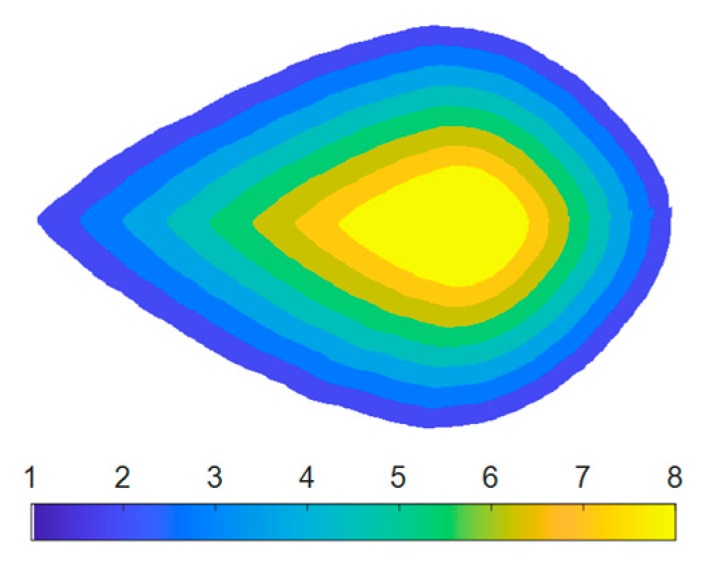
Eight sections on leaf image to detect the spatial variation.

Detecting color variance across the leaf is a significant advantage of LeafScope comparing with dot meters. Tirado et al. reported a simple method based on concentric rings to study the spatial variation across a corn plant [31]. Similarly, we evaluated LeafScop’s performance in detecting the color variation across the leaf on the computer. The NDVI image was used as an example product from the raw multispectral image in this study. Each leaf was divided into eight sections, as shown in Figure 6. The contour rings were obtained by scaling the leaf’s contour with 0.9, 0.8, 0.7, 0.6, 0.5, 0.4, and 0.3, respectively, with respect to a scaling center. The scaling center was selected on the midrib (major vein), which was approximately the symmetric axis of the soybean leaf. As the maximum value was approximately between the leaf center and base, the point at two thirds of the major vein from the leaf tip was selected as the scaling center. All pixels on each section were averaged for further analysis.

### 3.4. Leaf Venation Features Extraction

**Figure 7:**
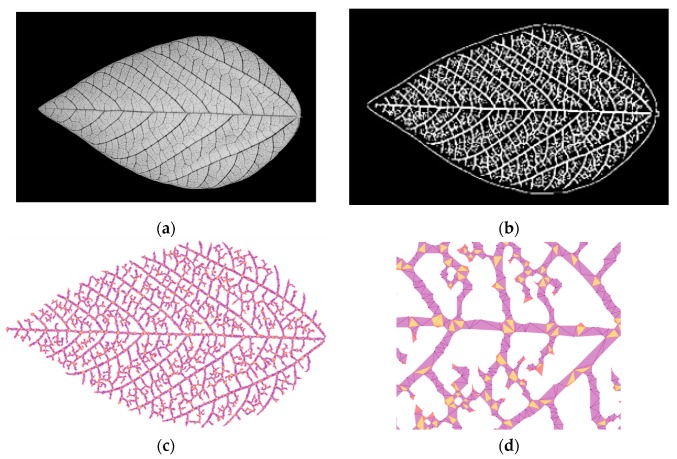
The extraction of leaf venation. (**a**) The NDVI image, (**b**) segmented leaf venation image, (**c**) extracted leaf venation image, (**d**) detail view of the leaf venation image (Yellow dots are junctions; pink dots are tips and purple curves are branches).

High-resolution leaf venation information is another advantage of LeafScope comparing with dot meters and other leaf imagers. We evaluated LeafScope by comparing leaf venation features in different treatments. In this study, leaf venation and its features were automatically extracted with the network extraction tool (NET) reported by Lasser et al. on the computer [12]. NET is a new method to automatically extract leaf venation features based on a high-resolution grayscale image, and is programmed in Python language and open-sourced on GitHub (https://github.com/JanaLasser/network_extraction). Due to the high contrast between veins and areoles, the NDVI image (Figure 7a) was selected as the input image for NET. With NET, the NDVI image was blurred with Gaussian smoothing with a kernel size of 5, segmented with an adaptive threshold algorithm with a block size of 51, and denoised by removing the small object less than 71 pixels. Then NET extracted the leaf venation graph (Figure 7c). Based on the graph, NET calculated the leaf venation features, including the number of junctions, number of endpoints, number of cycles, total length, total network area, area of the convex hull, average edge length, and average edge width [12].

### 3.5. Statistical Analysis

Averaged NDVI values, all of the morphological features and leaf venation features were analyzed with three-way analyses of variance (ANOVA) to detect the effect of the treatments on the plants. The mean values of all features were compared between all groups using the one-way ANOVA with a Tukey test. The effect of treatments on response variables was dealt with significance when P < 0.05. To evaluate the ability of measuring nitrogen content compared against the SPAD meter, a linear regression between averaged NDVI and averaged SPAD values was conducted. The averaged SPAD value of each leaf was calculated by averaging the six-dots measurements by SPAD meter. Furthermore, the averaged SPAD values were also predicted with the averaged intensities of all bands from LeafScope. The prediction model was obtained using the multiple linear regression (MLR) with interaction. When training the model, the leave-one-out cross-validation method was applied to avoid overfitting. The R^2^ value and RMSE were computed in the regression for evaluation.

## 4. Results and Discussions

### 4.1. Color Features

#### 4.1.1. Averaged Color Features of Whole Leaves

**Figure 8:**
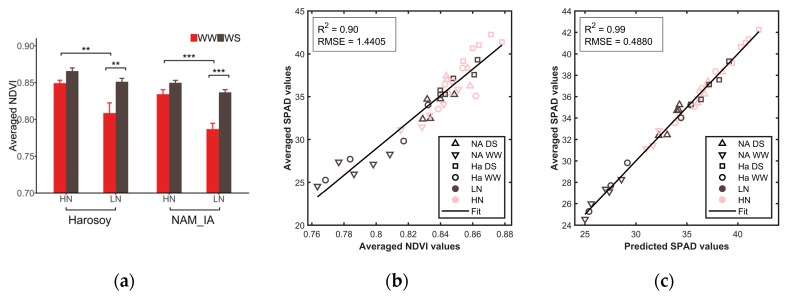
Averaged NDVI and averaged SPAD values of different groups. (**a**) Averaged NDVI by LeafScope for each leaf (P < 0.01 for genotype, P < 0.0001 for water, P < 0.0001 for N by three-way ANOVA). Data are represented as mean ±SE (n = 5). *P < 0.05, **P < 0.01, ***P < 0.001. (**b**) Relationship between averaged NDVI and averaged SPAD. (**c**) Relationship between predicted SPAD values by LeafScope and measured SPAD values by SPAD meter. DS for drought-stressed, WW for well-watered, LN for low nitrogen, HN for high nitrogen, NA for NAM_IA genotype, and Ha for Harsoy genotype in (**b**,**c**).

As Figure 8a shows, LeafScope detected the effect of genotype, water, and N treatments on the averaged NDVI values with P < 0.01. Moreover, the averaged NDVI values from Harosoy, HN and DS group were significantly higher than NAM_IA, LN and WW group, respectively. DS plants had significantly higher NDVI values than WW plants, especially for LN plants. The SPAD values also showed the same result. During severe drought stress, NDVI is expected to drop due to the loss of chlorophyll content. However, when drought stress is minor, it is possible to observe higher NDVI because the plants are growing slower, but they do not look stressed yet. The smaller plants would have lower requirements on nitrogen supply compared with the bigger canopy of WW plants; thus, they could look greener. More nutrients could also be washed away during the watering process for the well-watered plants, which were watered to excess. We did observe higher NDVI in some other greenhouse and field assays as well.

A linear regression model was fitted between the averaged NDVI and the averaged SPAD values. As Figure 8b shown, there was a close linear relationship between them with R^2^ of 0.90 and RMSE of 1.4405. Furthermore, we used the values of all bands to predict the SPAD values based on the MLR algorithm. Linear regression was fitted between predicted SPAD values by LeafScope and measured SPAD values by SPAD meter. As Figure 8c shows, LeafScope predicted SPAD values with higher accuracy (R^2^ = 0.99, RMSE = 0.4880). Thus, the results showed that LeafScope was capable of detecting the effect of genotype, water, and nitrogen treatments of soybean plants on the NDVI values. Moreover, in comparison with SPAD meter, LeafScope had the potential of measuring N content with high performance, especially when utilizing all color information of the leaf.

#### 4.1.2. Color Distribution across the Leaf

**Figure 9:**
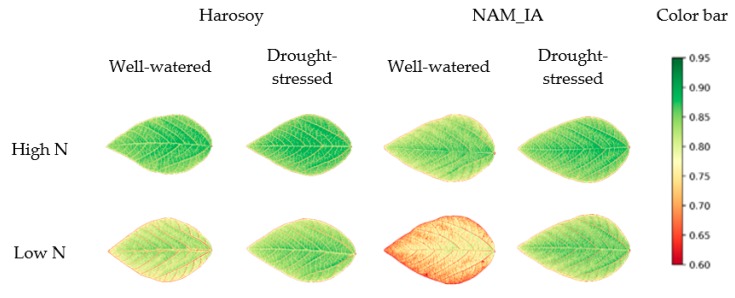
NDVI heatmap images of soybean leaves.

**Figure 10:**
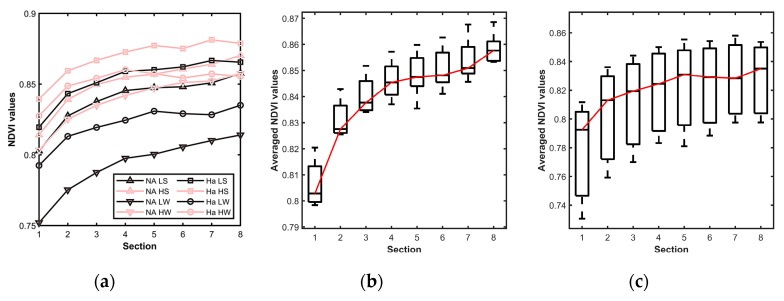
Distribution of NDVI values across soybean leaves. (**a**) NDVI values of each section of all groups, HS for high N and drought-stressed; HW for high N and well-watered; LS for low N and drought-stressed; LW for low N and well-watered, NA for NAM_IA genotype, and Ha for Harsoy genotype. (**b**) Averaged NDVI values of each section of drought-stressed and low N treated NAM_IA plants group (P < 0.0001 by one-way ANOVA). (**c**) Averaged NDVI values of each section of well-watered and low N treated Harsoy plants group (P = 0.176 by one-way ANOVA).

With the high-resolution multispectral images obtained by LeafScope, the color distribution on a leaf could be viewed clearly and analyzed. The images in Figure 9 show that the NDVI distribution across the leaf was not uniform. All the veins had relatively lower NDVI values, especially the major and first-order veins. Moreover, the pixels close to the major vein had higher NDVI values than the leaf edge. This pattern was proved by the contour scaling rings method. As Figure 10a shows, NDVI values of all treatments increased from leaf edge to the center. One-way ANOVA was applied to each treatment with respect to eight sections to test the NDVI distribution variation on the leaves. The results showed that the NDVI distribution was nonuniform (P < 0.01) in all treatments except WW × LN × Harsoy. The NDVI distribution of DS × LN × NAM_IA treatment showed the most significant variation with P < 0.0001 (Figure 10b), while that of WW × LN × Harsoy treatment was uniform with P = 0.176 (Figure 10c). Therefore, the results showed that LeafScope was able to detect the color variation across the soybean leaf, which could not be detected easily and precisely by dot meter or other leaf imagers. Moreover, the color variation enables us to study the difference between different sections on the leaf at the pixel level, and provides more signals to build a model of estimating plant physiology features (future work).

### 4.2. Leaf Morphological Features

After spatial calibration of the LeafScope camera, the 95% confidence interval of the absolute measuring errors in the imaging chamber is ±0.1627 mm. The relative measurement errors of all morphological features are less than 1.112% when the minor axis length of leaves is over 30 mm. Thus, the calibration accuracy is very high for measuring leaf morphological features. 

**Figure 11:**
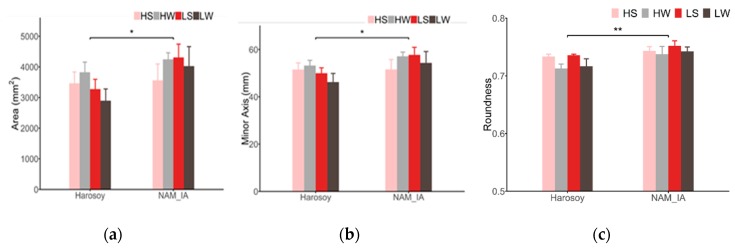
Leaf morphological features’ difference between groups by LeafScope. (**a**) Leaf area (P < 0.05 for genotype, P = 0.7477 for water, P = 0.6284 for N by three-way ANOVA); (**b**) Leaf minor axis length (P < 0.05 for genotype, P = 0.9747 for water, P = 0.5925 for N by three-way ANOVA); (**c**) Leaf roundness (P < 0.01 for genotype, P = 0.4251 for N, P < 0.05 for water, by three-way ANOVA). Data are represented as mean ±SE (n = 5). *P < 0.05, **P < 0.01 for genotype effect by three-way ANOVA. HS, high N and drought-stressed; HW, high N and well-watered; LS, low N and drought-stressed; LW, low N and well-watered.

To evaluate the LeafScope’s performance of detecting the effect of genotype, nitrogen, and water, three-way ANOVA was applied to all morphological features. The significant differences between genotypes were detected on area, minor axis, and roundness. As shown in Figure 11, leaves of genotype NAM_IA were significantly larger and rounder than that of Harosoy. Further, leaves of DS plants were significantly rounder than that of WW plants, but the difference of leaf area between WW plants and DS plants was not detected. The reason might be that the drought stress caused an adjustment of cell expansion and cell growth, which resulted in a rounder leaf, but the drought stress was not severe enough to reduce the leaf growth [32]. The results showed that LeafScope was able to provide high-quality data of the leaf’s morphological features.

The portable leaf area meter is one of the most commonly used devices to measure leaf morphological features in plant phenotyping. The meter, such as LI-3000C (LI-COR Biosciences, Lincoln, NE, USA) and CI-202 (CID Bio-Science, Inc., Camas, WA, USA) provides a method to in vivo measure the leaf morphological features, such as area, perimeter, and roundness. Comparing with these leaf area meters, LeafScope measures leaves without relative motion. No motion action applying on leaves means non-destruction for plants. Moreover, leaf images can provide more spatial information than leaf area meters, such as the leaf’s convex hull, which is useful for irregular leaf, and morphological features of leaf holes, which are useful in disease and pest detection.

### 4.3. Leaf Venation Features

**Figure 12:**
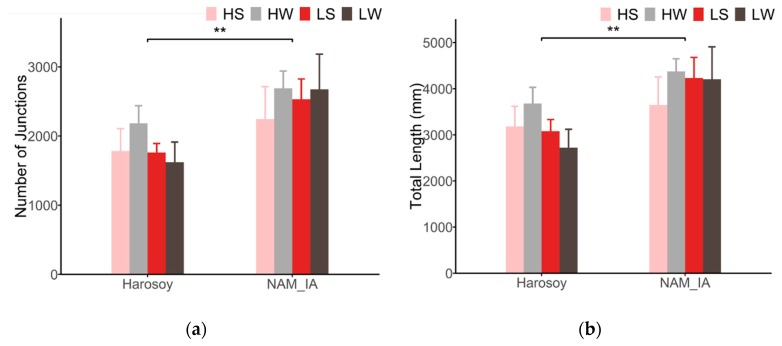
Leaf venation difference between groups. (**a**) Number of venation junctions (P < 0.01 for genotype, P = 0.746 for N, P = 0.378 for water by three-way ANOVA). (**b**) Total length of leaf venation (P < 0.01 for genotype, P = 0.618 for N, P = 0.524 for water by three-way ANOVA)). Data are represented as mean ±SE (n = 5). **P < 0.01 for genotype effect by three-way ANOVA. HS, high N and drought-stressed; HW, high N and well-watered; LS, low N and drought-stressed; LW, low N and well-watered.

LeafScope provides a new method to measure the leaf venation and study the relationship between leaf venation pattern and plant physiological activities. With high-resolution NDVI images obtained by LeafScope, leaf venation features could be extracted and analyzed. The experiment results showed that genotype impacts the number of vein junctions (nodes) and total veins length significantly (Figure 12). The genotype NAM_IA has more junctions and longer veins than Harosoy. No significant difference was detected in different N and water treatments. Therefore, leaf venation differences highly depended upon genotypes. In this publication, we were not able to collect ground truth measurements for the leaf venation. We will label the venation manually and then identify the venation net with deep learning next step. Then, more experiments will be conducted to study the leaf venation pattern.

The results showed that LeafScope was able to provide high-resolution leaf images to measure venation features and detect the effect of different treatments on it. Currently, to obtain leaf venation images, leaves are imaged after harvest or with an X-ray device which is expensive and not suitable for field plants [11,15,33]. Comparing with these imaging systems, LeafScope suggested a low-cost method to image leaf venation in vivo.

### 4.4. Opportunities for Future Work

In this study, we developed LeafScope, which could detect nitrogen status, morphological features, and leaf venation patterns with the validation of the experiment in the greenhouse. Next, we will optimize it with field tests. There are three directions for the optimization work. First, for field application, there might be more dust than that in the greenhouse; thus, the mechanism of auto cleaning the imaging chamber might be required. Second, plant stress in the field is more complicated than that in the greenhouse. Thus, the model of detecting different stresses such as nitrogen, water, disease, and pest will be studied and specified for LeafScope. Third, measuring leaf manually one by one is labor-consuming, especially in the field. Thus, we will develop a field robot to manipulate LeafScope to image soybean leaf automatically.

LeafScope is developed for dicotyledon plants. Leaves of different species are diverse in morphological features and leaf venation. We will test LeafScope on other species to evaluate its performance, such as apple leaf. In this publication, the spatial variation of color across leaves was proved. New algorithms will be developed based on the variation to improve the quality of estimating plant physiological features. Moreover, we will also apply LeafScope to track the changes in leaf venation along with different growth stages.

## 5. Conclusions

A new, portable multispectral imaging device, LeafScope, was developed for the high-quality and non-destructive multispectral imaging of dicotyledon plants leaves. The device provides a new method to in vivo monitor plant health by capturing multispectral leaf images. It provides color information from visible to NIR, morphological features, and sub-leaf-level features, such as color distribution across the leaf and venation pattern.

To evaluate the performance of the device, the greenhouse assay test was conducted on forty soybean plants with different genotypes, nitrogen, and water treatments. The color, morphology, and venation features of each leaf were extracted for analysis. The experiment results showed: (1) The effect of genotype, nitrogen, and water on NDVI were clearly detected by LeafScope (P < 0.01). (2) The SPAD values were predicted with high accuracy by LeafScope (R^2^ = 0.99, RMSE = 0.4735). 3) LeafScope detected that the NDVI values in different sections on leaves were significantly different (P < 0.001) for all treatments except WW × LN × Harsoy. 4) The device also enabled us to measure morphological and venation features. LeafScope detected the significant difference between different genotypes with respect to leaf morphological features, such as area (P < 0.05), and leaf venation features, such as venation nodes (P < 0.05). Moreover, with LeafScope, leaves could be measured and studied in time series, as the measurement does not damage the plant. So, the measurement could be repeated in time series. Further research will be conducted to develop new models of predicting plant physiological features based on the multispectral images of whole leaves with LeafScope.

## Figures and Tables

**Table 1:** Specifications of the LeafScope components.

Component	Price ($)	Parameters	Values
Camera	519	Model	BFLY-U3-23S6M-C
		Resolution	1920 × 1200
		Frame rate	41 FPS
		Chroma	Mono
		Sensor mode	Sony IMX249
		Pixel size	5.86 µm
		Shutter type	Global
		ADC	12 bit
		Dynamic range (dB)	67.12
		Dimensions (mm)	29 × 29 × 30
		Mass (g)	36
Lens	379	Model	V0814-MP
		Focal length	8 mm
LED 405 nm	24	FWHM	40 nm
LED 560 nm	10	FWHM	24 nm
LED 660 nm	5	FWHM	38 nm
LED 860 nm	14	FWHM	50 nm
Controller	49	Model	ODROID XU4

Note: FWHM is full width at half maximum.
